# Overexpression of MEKK2 is associated with colorectal carcinogenesis

**DOI:** 10.3892/ol.2013.1553

**Published:** 2013-08-29

**Authors:** LI JIANG, MEIJIN HUANG, LEI WANG, XINJUAN FAN, PUNING WANG, DAOHAI WANG, XINHUI FU, JIANPING WANG

**Affiliations:** 1 Department of Gastrointestinal Surgery, Shenzhen People’s Hospital, Shenzhen, Guangdong 518020, P.R. China; 2 Department of Colorectal Surgery, The Sixth Affiliated Hospital of Sun Yat-sen University, Guangzhou, Guangdong 510655, P.R. China; 3 Department of General Surgery, Henan Cancer Hospital, Zhengzhou, Henan 450008, P.R. China

**Keywords:** immunohistochemistry, colorectal cancer, MEKK2, carcinogenesis

## Abstract

Mitogen-activated protein kinase kinase kinase 2 (MEKK2) is an important upstream mediator of the extracellular signal-regulated kinase 5 signaling cascade that is essential for a number of cellular functions, including mitogenesis, differentiation and oncogenic transformation. Using western blotting to examine MEKK2 expression in 16 cases of primary colorectal cancer (CRC) lesions with paired normal mucosa, it was identified that MEKK2 is highly expressed in CRC lesions compared with that of the normal mucosa. Immunohistochemistry of 24 normal mucosa, 24 adenoma and 96 adenocarcinoma colorectal specimens indicated that the expression of MEKK2 was significantly increased in the adenoma and carcinoma specimens compared with that of the normal mucosa cases (P<0.0001 for both). However, no significant differences were detected in MEKK2 expression between the carcinoma and adenoma specimens (P=0.85). Similarly, no correlations were identified between MEKK2 expression and clinicopathological features, including gender, age, body mass index, histological differentiation, depth of invasion, lymph node metastasis, UICC stage and K-ras mutations (P>0.05). The present study demonstrated that MEKK2 functions as a promotive factor in CRC.

## Introduction

Colorectal cancer (CRC) is the second and third most common type of malignancy in females and males worldwide, respectively (1). In the majority of Asian countries, CRC morbidity has increased rapidly throughout previous decades, and follows a stepwise progression from normal tissue to a premalignant phase to the invasive carcinoma. This process is known as the adenoma-carcinoma sequence. Recently, a number of studies have suggested that the accumulation of multiple gene mutations in key signaling pathways correlates with the multiple steps of colorectal carcinogenesis (2,3). Therefore, it is essential to identify significant molecular biomarkers in these pathways for the prevention and treatment of CRC.

Mitogen-activated protein kinase kinase kinase 2 (MEKK2) is a Ser/Thr protein kinase expressed in multiple tissues that belongs to the MEKK/STE11 subgroup of the MAP3K family (4,5). Specifically, MEKK2 is a member of the extracellular signal-regulated kinase 5 (ERK5) signaling cascade that has been previously identified in all four mitogen-activated protein kinase (MAPK) pathways, including MEKK2/3, MEK5 and ERK5 at the MAP3K, MAPKK and MAPK tiers, respectively (6). The ERK5 signaling cascade is essential for the control of cellular proliferation (7) and is likely to be targeted during cell cycle progression (8,9) and tumorigenesis (10,11). In addition, the ERK5 cascade is involved in the management of cell differentiation (12,13), migration (14,15), neuronal survival rate (16), embryonic angiogenesis (17) and additional cellular functions (18–21).

MEKK2 has been shown to mediate epidermal growth factor and fibroblast growth factor 2 receptor signals (22,23) which have been previously identified to be involved in the development of various types of cancer (24,25). Therefore, MEKK2 may also be important for the development of cancer. However, although overexpression of MEKK2 has been identified in prostate cancer (26), to the best of our knowledge, no studies have analyzed the expression of MEKK2 in CRC. Therefore, the aim of the present study was to determine MEKK2 expression at the protein level in colorectal carcinomas and adenomas, as well as normal epithelial specimens, by immunohistochemistry (IHC), to explore the significance of MEKK2 in the development of CRC. The potential correlation between MEKK2 expression and the major clinicopathological features of CRC and K-ras mutations was also assessed to determine the novel involvement of MEKK2 in the malignant development of CRC.

## Materials and methods

### Western blot analysis

Sixteen pairs of randomly collected CRC patient and matched non-cancerous tissue specimens were analyzed by western blot analysis. Total protein extracted from frozen colorectal mucosa was resolved using 12% SDS polyacrylamide gel and electrotransferred to a polyvinylidene difluoride membrane (Pall, Port Washington, NY, USA). Following blocking with 5% bovine serum albumin for 1 h, the tissues were incubated with primary rabbit monoclonal antibody against MEKK2 (EP626Y; Abcam, Cambridge, UK) at a dilution of 1:10,000. The immunoreactive bands were detected using enhanced chemiluminescence reagents (GE Healthcare, Uppsala, Sweden). The procedures were conducted according to the manufacturer’s instructions and β-actin was used as a loading control.

### Tissue samples

Specimens were randomly selected from archival tissues surgically extracted at the Sixth Affiliated Hospital of Sun Yat-sen University (Guangzhou, China) between 2009 and 2010. Tissue specimens from the local pathology repository, as well as clinical data were available. Formalin-fixed paraffin-embedded specimens were collected for IHC analysis, including 24 normal epithelial, 24 adenoma and 96 primary adenocarcinoma colorectal specimens, classified as stages I (n=16), II (n=23), III (n=43) and IV (n=14), according to the International Union Against Cancer tumor-node-metastasis (TNM) staging criteria (7^th^ version, 2009). The patient group included 80 males and 64 females with a mean age of 60 years (range, 18–87 years). The present study was approved by the ethics committee of The Sixth Affiliated Hospital of Sun Yat-sen University (Guangzhou, China) and written informed consent was obtained from each participant.

### Tissue microarray

Hematoxylin and eosin-stained slides from each tumor block were examined to select a morphologically representative area. Two core tissue biopsies with a diameter of 1 mm were punched from the marked area of each donor block and transferred to the recipient paraffin blocks using the precise Minicore^®^ Tissue Arrayer (Alphelys, Plaisir, France). Three tissue microarrays were constructed, including 8 normal epithelial, 8 adenoma and 32 carcinoma colorectal specimens. The tissue microarray blocks were cut into 4 μm-sections using a microtome, mounted on polylysine-coated glass slides and used for IHC analysis.

### IHC

Slides were deparaffinized in xylene and rehydrated using a graded ethanol series. Antigens were retrieved by boiling the slides in a microwave oven for 15 min in 0.01 mol/l citrate buffer (pH 6.0). Endogenous peroxidase was blocked with 0.3% hydrogen peroxide solution and the slides were incubated in 10% normal goat serum for 15 min to prevent nonspecific staining. The tissue sections were then incubated overnight at 4°C with rabbit monoclonal antibody against MEKK2 (EP626Y; Abcam) at a dilution of 1:200. Subsequently, the standard horseradish peroxidase/DAB (Dako Envision+ System; Dako, Carpinteria, CA, USA) method was used and the slides were lightly counterstained with hematoxylin. Positively stained CRC sections were included as negative and positive controls, and sections incubated with antibody diluent instead of the primary antibody were used as negative controls.

### Evaluation of IHC

Immunohistochemical staining was examined and evaluated by two independent pathologists. The staining results were scored according to the intensity of staining: 0, negative; 1, bordering; 2, weak; 3, moderate and 4, strong. In addition, the percentage of tumor cells stained was scored: 0, none; 1, 1–25%; 2, 26–50%; 3, 51–75% and 4, 76–100%. Staining index was calculated as follows: staining index = staining intensity score × proportion score. Using this method, the expression of MEKK2 in cores was evaluated by determining the staining index and scores were arranged between 0 and 16. Each specimen provided two cores and the score of each specimen was acquired from their mean values. Tumor specimens were grouped into two categories defined as follows: i) high expression, with an average score of ≥8; and ii) low expression, with an average score of <8.

### Statistical analysis

Statistical analyses were performed using SPSS 17.0 for Windows (SPSS, Inc., Chicago, IL, USA). The χ^2^, Kruskal-Wallis or Mann-Whitney U tests were used to analyze the correlation between MEKK2 expression in normal mucosa, adenoma and carcinoma specimens and clinicopathological features. All P-values are two-sided and P<0.05 was considered to indicate a statistically significant difference.

## Results

### MEKK2 protein expression levels in CRC and normal colorectal tissues

**Figure 1:**
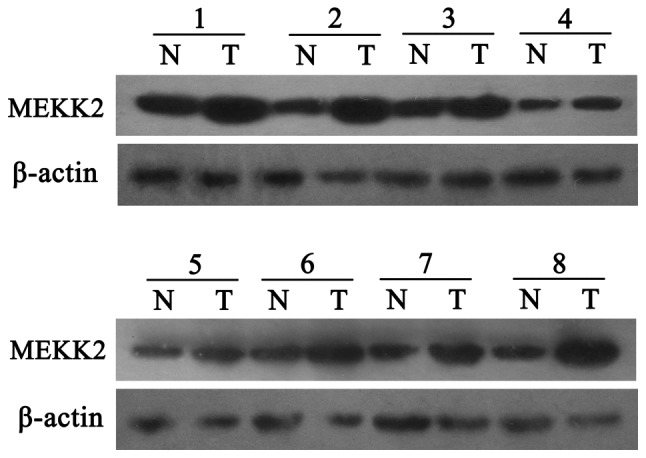
Western blot analysis of MEKK2 protein expressed in eight pairs of colorectal tumor (T) and matched nontumor tissues (N). Expression levels were normalized against β-actin. MEKK2, mitogen-activated protein kinase kinase kinase 2.

Expression of MEKK2 protein was primarily investigated by western blot analysis in 16 randomly selected pairs of CRC and matched non-cancerous colorectal tissues. MEKK2 protein was overexpressed in 13 (81.3%) tumor tissues, which was significantly higher than that of non-cancerous colorectal tissues (Fig. 1).

### MEKK2 expression in adjacent normal mucosa, adenoma and carcinoma

**Figure 2:**
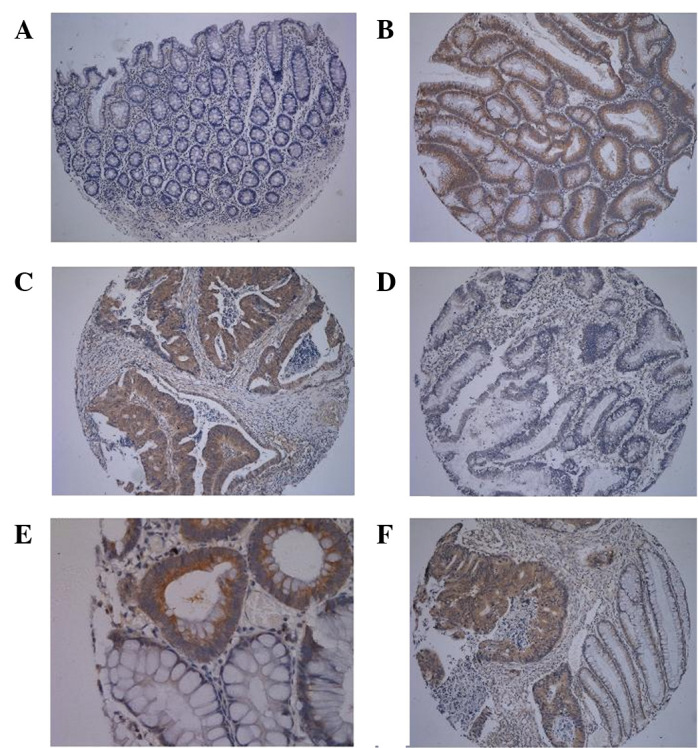
Immunohistochemical staining of MEKK2 in adjacent normal mucosa, adenoma and adenocarcinoma of the colorectum. (A) Low expression in normal mucosa, (B) high expression in adenoma, (C) high and (D) low expression in carcinoma and (E) higher expression in adenoma and (F) carcinoma, compared with that of neighboring normal mucosa. MEKK2, mitogen-activated protein kinase kinase kinase 2.

MEKK2 expression was examined by IHC in 24 adjacent normal mucosa, 24 adenoma and 96 carcinoma specimens. Expression was detected in the cytoplasm of normal mucosa and tumor epithelial cells (Fig. 2), but no nuclear staining was identified. Low expression of MEKK2 was identified in all 24 normal mucosa specimens (100%), with no detection of high expression (0%). In the adenomas, 18 (75.0%) specimens exhibited strong expression and 6 (25.0%) exhibited weak expression. In the carcinomas, 80 (83.3%) specimens exhibited strong expression and 16 (16.7%) exhibited weak expression.

A significant difference was identified in the overall distribution of MEKK2 expression among the normal mucosa, adenoma and carcinoma specimens (χ^2^=61.97; P<0.001; Table I). High expression of MEKK2 was significantly increased in adenoma (P<0.0001) and carcinoma specimens when compared with that of the normal mucosa specimens (P<0.0001). However, although expression was increased in the adenoma specimens compared with the carcinomas, MEKK2 levels were not found to be significantly different (P=0.85).

### Correlation between MEKK2 expression and clinicopathological variables in carcinomas

The correlation between MEKK2 expression in carcinomas and a set of clinicopathological variables were analyzed. However, no significant correlations were observed between MEKK2 expression and clinicopathological variables, including gender, age, body mass index, histological differentiation, depth of invasion, lymph node metastasis and UICC stage (P>0.05; Table II).

### Correlation between MEKK2 expression and K-ras mutations in adenomas and carcinomas

In addition, the correlation between MEKK2 expression and the K-ras mutation state in carcinomas was analyzed, however, no correlation was identified (P=0.68; Table II).

## Discussion

The majority of CRCs arise via the adenoma-carcinoma sequence. A number of molecular and pathway alterations have been identified during this process, which are specific to the transformation from normal cells to adenoma, development from adenoma to cancer or the process as a whole (2,3). Analyzing the alterations of molecular biomarkers may aid in the prevention, early diagnosis and treatment of CRC. In the current study, the correlation between MEKK2 expression and clinicopathological features was investigated in various CRC tissues for the first time. The results demonstrate that MEKK2 may be involved in the development of CRC.

As one of the only two upstream molecules in the ERK5 cascade, MEKK2 is crucial in relaying specific cell surface signals to downstream ERK5 following transcription regulation. MEKK2 is distributed in the cytoplasm and forms a complex with the MEKK2-interacting protein, which prevents its activation (27). When cells are stimulated by the epidermal growth factor (EGF) and additional stimuli, the complex dissociates and the number of MEKK2 molecules increases (27). Overexpression of MEKK2 is sufficient for the formation of dimers leading to self-phosphorylation and activation (28). In the present study, free MEKK2 protein was detected in various colorectal tissues, indicating a correlation with the development of CRC. MEKK2 was notably upregulated during the transition from normal epidermal to adenoma, and from adenoma to carcinoma. A significant difference was identified in MEKK2 expression levels between adenoma and carcinoma, indicating that MEKK2 may represent a promotive factor of CRC, which is important for the early stages of CRC development. In addition, inhibition of MEKK2 expression may block the development of CRC and therefore, be significant for the development of colorectal adenoma.

However, in the present study, no correlations were identified between MEKK2 expression and the critical clinicopathological factors of carcinoma, including TNM stage, degree of differentiation and in particular, indicators of CRC foci-invasive and metastatic dissemination capabilities. This indicates that the correlation between MEKK2 expression and CRC carcinogenesis is negligible; however, MEKK2 may not be excluded as a biomarker for adenoma-carcinoma progression. Similarly, no significant correlation was identified between the expression of MEKK2 and K-ras mutation status. These observations are in accordance with a study by Kato *et al,* which reported that EGF-mediated activation of ERK5 proceeds independently of Ras (7).

Currently, the mechanisms responsible for the correlation between MEKK2 expression and the neoplastic process are not yet fully understood and require investigation. The significance of this correlation may be examined via the downstream molecule ERK5, which has been investigated in a number of previous studies. ERK5 functions as a survival rate factor in mitosis (9), and is essential for EGF-mediated cell proliferation and for cells to enter the S phase of the cell cycle (10). In addition, overexpression of ERK5 has been identified in various types of cancer, including breast and prostate cancer and oral squamous cell carcinoma, and has been hypothesized to represent an independent prognostic biomarker of disease-free survival (11,29,30). Therefore, overexpression of MEKK2, as an important upstream mediator, activates ERK5 and initiates the development of CRC.

A limitation of the present study was that the patients were newly diagnosed with CRC in the last two years and were deficient of prognostic data. Therefore, the prognostic value of MEKK2 in CRC has not been assessed and requires investigation in future studies.

In conclusion, the results of the current study indicate that overexpression of MEKK2 correlates with colorectal carcinogenesis and may represent a promotive factor in the development of CRC. Specifically, MEKK2 may be particularly important in the early phases of this stepwise process during the transition from normal epithelium to adenoma. However, future studies must investigate this correlation in detail to evaluate its application.

## Figures and Tables

**Table I:** Correlation between MEKK2 expression levels and various colorectal tissues.

		Low expression		High expression		
				
	n	n	%	n	%	P-value
n	96					
Normal mucosa	24	24	100.0	0	0.0	<0.001
Adenoma	24	6	25.0	18	75.0	
Carcinoma	96	16	16.7	80	83.3	

MEKK2, mitogen-activated protein kinase kinase kinase 2.

**Table II:** Correlation between MEKK2 expression levels and clinicopathological variables of colorectal carcinoma.

		Low expression		High expression		
				
Valuables	n	n	%	n	%	P-value
n	96					
Gender						0.78
Male	45	7	15.6	38	84.4	
Female	51	9	17.6	42	82.4	
Age, years-old						1.00
<55	30	5	16.7	25	83.3	
≥55	66	11	16.7	55	83.3	
BMI						0.19
<24.0	67	9	13.4	58	86.6	
≥24.0	29	7	24.1	22	75.9	
Histological differentiation						0.49
Well	22	4	18.2	18	81.8	
Moderate	63	9	14.3	54	85.7	
Poor	11	3	27.3	8	72.7	
T stage						0.75
1	7	1	14.3	6	85.7	
2	17	4	23.5	13	76.5	
3	57	8	14.0	49	86.0	
4	15	3	20.0	12	80.0	
N stage						0.27
0	42	9	21.4	33	78.6	
1–2	54	7	13.0	47	87.0	
UICC stage						0.42
I	16	5	31.3	11	68.8	
II	23	3	13.0	20	87.0	
III	43	6	14.0	37	86.0	
IV	14	2	14.3	12	85.7	
K-ras mutation						0.68
Yes	26	5	19.2	21	80.8	
No	70	11	15.7	59	84.3	

MEKK2, mitogen-activated protein kinase kinase kinase 2; BMI, body mass index; T stage, depth of invasion; N stage, lymph node metastasis.

## References

[1] Jemal A, Bray F, Center MM, Ferlay J, Ward E, Forman D (2011). Global cancer statistics. CA Cancer J Clin.

[2] Fearon ER, Vogelstein B (1990). A genetic model for colorectal tumorigenesis. Cell.

[3] Vogelstein B, Fearon ER, Hamilton SR (1988). Genetic alterations during colorectal-tumor development. N Engl J Med.

[4] Blank JL, Gerwins P, Elliott EM, Sather S, Johnson GL (1996). Molecular cloning of mitogen-activated protein/ERK kinase kinases (MEKK) 2 and 3. Regulation of sequential phosphorylation pathways involving mitogen-activated protein kinase and c-Jun kinase. J Biol Chem.

[5] Su B, Cheng J, Yang J, Guo Z (2001). MEKK2 is required for T-cell receptor signals in JNK activation and interleukin-2 gene expression. J Biol Chem.

[6] Yao Z, Yoon S, Kalie E, Raviv Z, Seger R (2010). Calcium regulation of EGF-induced ERK5 activation: role of Lad1-MEKK2 interaction. PLoS One.

[7] Kato Y, Tapping RI, Huang S, Watson MH, Ulevitch RJ, Lee JD (1998). Bmk1/Erk5 is required for cell proliferation induced by epidermal growth factor. Nature.

[8] Cude K, Wang Y, Choi HJ, Hsuan SL, Zhang H, Wang CY, Xia Z (2007). Regulation of the G2-M cell cycle progression by the ERK5-NFkappaB signaling pathway. J Cell Biol.

[9] Gírio A, Montero JC, Pandiella A, Chatterjee S (2007). Erk5 is activated and acts as a survival factor in mitosis. Cell Signal.

[10] Pearson G, English JM, White MA, Cobb MH (2001). ERK5 and ERK2 cooperate to regulate NF-kappaB and cell transformation. J Biol Chem.

[11] Montero JC, Ocaña A, Abad M, Ortiz-Ruiz MJ, Pandiella A, Esparís-Ogando A (2009). Expression of Erk5 in early stage breast cancer and association with disease free survival identifies this kinase as a potential therapeutic target. PLoS One.

[12] Carter EJ, Cosgrove RA, Gonzalez I, Eisemann JH, Lovett FA, Cobb LJ, Pell JM (2009). MEK5 and ERK5 are mediators of the pro-myogenic actions of IGF-2. J Cell Sci.

[13] Dinev D, Jordan BW, Neufeld B, Lee JD, Lindemann D, Rapp UR, Ludwig S (2001). Extracellular signal regulated kinase 5 (ERK5) is required for the differentiation of muscle cells. EMBO Rep.

[14] Schramp M, Ying O, Kim TY, Martin GS (2008). ERK5 promotes Src-induced podosome formation by limiting Rho activation. J Cell Biol.

[15] Spiering D, Schmolke M, Ohnesorge N (2009). MEK5/ERK5 signaling modulates endothelial cell migration and focal contact turnover. J Biol Chem.

[16] Watson FL, Heerssen HM, Bhattacharyya A, Klesse L, Lin MZ, Segal RA (2001). Neurotrophins use the Erk5 pathway to mediate a retrograde survival response. Nat Neurosci.

[17] Sohn SJ, Sarvis BK, Cado D, Winoto A (2002). ERK5 MAPK regulates embryonic angiogenesis and acts as a hypoxia-sensitive repressor of vascular endothelial growth factor expression. J Biol Chem.

[18] Nicol RL, Frey N, Pearson G, Cobb M, Richardson J, Olson EN (2001). Activated MEK5 induces serial assembly of sarcomeres and eccentric cardiac hypertrophy. EMBO J.

[19] Liu L, Cundiff P, Abel G (2006). Extracellular signal-regulated kinase (ERK) 5 is necessary and sufficient to specify cortical neuronal fate. Proc Natl Acad Sci USA.

[20] Nishimoto S, Nishida E (2006). MAPK signalling: ERK5 versus ERK1/2. EMBO Rep.

[21] Wang X, Tournier C (2006). Regulation of cellular functions by the ERK5 signalling pathway. Cell Signal.

[22] Sun W, Wei X, Kesavan K (2003). MEK kinase 2 and the adaptor protein Lad regulate extracellular signal-regulated kinase 5 activation by epidermal growth factor via Src. Mol Cell Biol.

[23] Kesavan K, Lobel-Rice K, Sun W, Lapadat R, Webb S, Johnson GL, Garrington TP (2004). MEKK2 regulates the coordinate activation of ERK5 and JNK in response to FGF-2 in fibroblasts. J Cell Physiol.

[24] Herbst RS, Shin DM (2002). Monoclonal antibodies to target epidermal growth factor receptor-positive tumors: a new paradigm for cancer therapy. Cancer.

[25] Billottet C, Elkhatib N, Thiery JP, Jouanneau J (2004). Targets of fibroblast growth factor 1 (FGF-1) and FGF-2 signaling involved in the invasive and tumorigenic behavior of carcinoma cells. Mol Biol Cell.

[26] Cazares LH, Troyer D, Mendrinos S (2009). Imaging mass spectrometry of a specific fragment of mitogen-activated protein kinase/extracellular signal-regulated kinase kinase kinase 2 discriminates cancer from uninvolved prostate tissue. Clin Cancer Res.

[27] Cheng J, Zhang D, Kim K, Zhao Y, Zhao Y, Su B (2005). Mip1, an MEKK2-interacting protein, controls MEKK2 dimerization and activation. Mol Cell Biol.

[28] Cheng J, Yu L, Zhang D, Huang Q, Spencer D, Su B (2005). Dimerization through the catalytic domain is essential for MEKK2 activation. J Biol Chem.

[29] McCracken SR, Ramsay A, Heer R (2008). Aberrant expression of extracellular signal-regulated kinase 5 in human prostate cancer. Oncogene.

[30] Sticht C, Freier K, Knöpfle K (2008). Activation of MAP kinase signaling through ERK5 but not ERK1 expression is associated with lymph node metastases in oral squamous cell carcinoma (OSCC). Neoplasia.

